# (*E*)-3-Bromo-*N*′-(2-chloro­benzyl­idene)benzohydrazide

**DOI:** 10.1107/S160053680902412X

**Published:** 2009-06-27

**Authors:** Lan-Zhu Qu, Guo-Biao Cao

**Affiliations:** a Department of Chemistry, Ankang University, Ankang Shanxi 725000, People’s Republic of China

## Abstract

The title compound, C_14_H_10_BrClN_2_O, was synthesized by the reaction of 2-chloro­benzaldehyde with an equimolar quantity of 3-bromo­benzohydrazide in methanol. The mol­ecule displays an *E* configuration about the C=N bond. The dihedral angle between the two benzene rings is 13.0 (2)°. In the crystal structure, mol­ecules are linked through inter­molecular N—H⋯O hydrogen bonds, forming chains propagating along the *c* axis.

## Related literature

For the crystal structures of hydrazone compounds, see: Mohd Lair *et al.* (2009 ▶); Fun *et al.* (2008 ▶); Li & Ban (2009 ▶); Zhu *et al.* (2009 ▶); Yang (2007 ▶); You *et al.* (2008 ▶). For hydrazone compounds reported previously by our group, see: Qu *et al.* (2008 ▶); Yang *et al.* (2008 ▶); Cao & Lu (2009*a* ▶,*b* ▶).
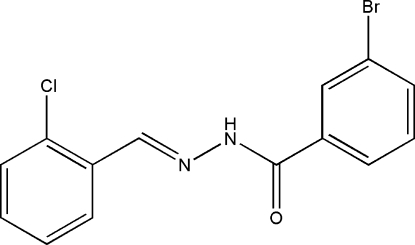


## Experimental

### 

#### Crystal data

C_14_H_10_BrClN_2_O
*M* *_r_* = 337.60
Monoclinic, 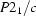

*a* = 13.140 (1) Å
*b* = 12.632 (1) Å
*c* = 8.377 (1) Å
β = 98.174 (2)°
*V* = 1376.3 (2) Å^3^
*Z* = 4
Mo *K*α radiation
μ = 3.17 mm^−1^
*T* = 298 K
0.27 × 0.25 × 0.22 mm

#### Data collection

Bruker SMART CCD area-detector diffractometer
Absorption correction: multi-scan (*SADABS*; Bruker, 2001 ▶) *T*_min_ = 0.481, *T*_max_ = 0.542 (expected range = 0.442–0.498)
8319 measured reflections
2998 independent reflections
2235 reflections with *I* > 2σ(*I*)
*R*_int_ = 0.025

#### Refinement

*R*[*F* ^2^ > 2σ(*F* ^2^)] = 0.033
*wR*(*F* ^2^) = 0.076
*S* = 1.03
2998 reflections
176 parameters
1 restraint
H atoms treated by a mixture of independent and constrained refinement
Δρ_max_ = 0.52 e Å^−3^
Δρ_min_ = −0.65 e Å^−3^

### 

Data collection: *SMART* (Bruker, 2007 ▶); cell refinement: *SAINT* (Bruker, 2007 ▶); data reduction: *SAINT*; program(s) used to solve structure: *SHELXTL* (Sheldrick, 2008 ▶); program(s) used to refine structure: *SHELXTL*; molecular graphics: *SHELXTL*; software used to prepare material for publication: *SHELXTL*.

## Supplementary Material

Crystal structure: contains datablocks global, I. DOI: 10.1107/S160053680902412X/ci2833sup1.cif

Structure factors: contains datablocks I. DOI: 10.1107/S160053680902412X/ci2833Isup2.hkl

Additional supplementary materials:  crystallographic information; 3D view; checkCIF report

## Figures and Tables

**Table 1:** Hydrogen-bond geometry (Å, °)

*D*—H⋯*A*	*D*—H	H⋯*A*	*D*⋯*A*	*D*—H⋯*A*
N2—H2⋯O1^i^	0.89 (1)	1.98 (1)	2.854 (2)	165 (3)

Symmetry code: (i) 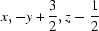.

**Fig. 1.**
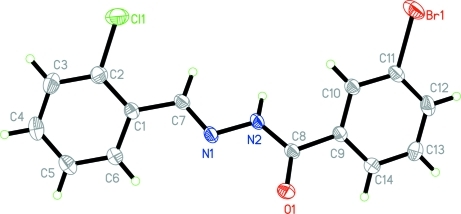
The molecular structure of the title compound. Displacement ellipsoids are drawn at the 30% probability level.

**Fig. 2.**
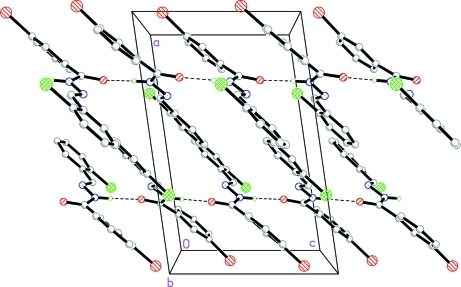
The crystal packing of the title compound, viewed along the b axis. H atoms not involved in hydrogen bonding (dashed lines) have been omitted for clarity.

## supplementary crystallographic information

### Comment

Study on the crystal structures of hydrazone derivatives is a hot topic in structural chemistry. In the last few years, the crystal structures of a large number of hydrazone compounds have been reported (Mohd Lair *et al.*, 2009; Fun *et al.*, 2008; Li & Ban, 2009; Zhu *et al.*, 2009; Yang, 2007; You *et al.*, 2008). As a continuation of our work in this area (Qu *et al.*, 2008; Yang *et al.*, 2008; Cao & Lu, 2009a,b), the title new hydrazone compound derived from the reaction of 2-chlorobenzaldehyde with an equimolar quantity of 3-bromobenzohydrazide is reported.

In the title compound (Fig. 1), the dihedral angle between the two benzene rings is 13.0 (2)°. The molecule displays an *E* configuration about the C═N bond. In the crystal structure, molecules are linked through intermolecular N—H···O hydrogen bonds (Table 1) to form chains running along the *c* axis (Fig. 2).

### Experimental

The title compound was prepared by refluxing equimolar quantities of 2-chlorobenzaldehyde with 3-bromobenzohydrazide in methanol. Colourless block-like crystals were formed by slow evaporation of the solution in air.

### Refinement

Atom H2 was located in a difference Fourier map and refined isotropically, with the N-H distance restrained to 0.90 (1) Å. The other H atoms were placed in idealized positions and constrained to ride on their parent atoms, with C-H distances of 0.93 Å, and with *U*_iso_(H) set at 1.2*U*_eq_(C).

### Crystal data

**Table:**

C_14_H_10_BrClN_2_O	*F*(000) = 672
*M**_r_* = 337.60	*D*_x_ = 1.629 Mg m^−^^3^
Monoclinic, *P*2_1_/*c*	Mo *K*α radiation, λ = 0.71073 Å
Hall symbol: -P 2ybc	Cell parameters from 2981 reflections
*a* = 13.140 (1) Å	θ = 2.2–27.5°
*b* = 12.632 (1) Å	µ = 3.17 mm^−^^1^
*c* = 8.377 (1) Å	*T* = 298 K
β = 98.174 (2)°	Block, colourless
*V* = 1376.3 (2) Å^3^	0.27 × 0.25 × 0.22 mm
*Z* = 4	

### Data collection

**Table:**

Bruker SMART CCD area-detector diffractometer	2998 independent reflections
Radiation source: fine-focus sealed tube	2235 reflections with *I* > 2σ(*I*)
graphite	*R*_int_ = 0.025
ω scans	θ_max_ = 27.0°, θ_min_ = 1.6°
Absorption correction: multi-scan (*SADABS*; Bruker, 2001)	*h* = −16→15
*T*_min_ = 0.481, *T*_max_ = 0.542	*k* = −16→13
8319 measured reflections	*l* = −10→10

### Refinement

**Table:**

Refinement on *F*^2^	Secondary atom site location: difference Fourier map
Least-squares matrix: full	Hydrogen site location: inferred from neighbouring sites
*R*[*F*^2^ > 2σ(*F*^2^)] = 0.033	H atoms treated by a mixture of independent and constrained refinement
*wR*(*F*^2^) = 0.076	*w* = 1/[σ^2^(*F*_o_^2^) + (0.0266*P*)^2^ + 0.7889*P*] where *P* = (*F*_o_^2^ + 2*F*_c_^2^)/3
*S* = 1.03	(Δ/σ)_max_ = 0.001
2998 reflections	Δρ_max_ = 0.52 e Å^−^^3^
176 parameters	Δρ_min_ = −0.65 e Å^−^^3^
1 restraint	Extinction correction: *SHELXTL* (Sheldrick, 2008), Fc^*^=kFc[1+0.001xFc^2^λ^3^/sin(2θ)]^-1/4^
Primary atom site location: structure-invariant direct methods	Extinction coefficient: 0.0155 (8)

### Special details

**Table:**

Geometry. All esds (except the esd in the dihedral angle between two l.s. planes) are estimated using the full covariance matrix. The cell esds are taken into account individually in the estimation of esds in distances, angles and torsion angles; correlations between esds in cell parameters are only used when they are defined by crystal symmetry. An approximate (isotropic) treatment of cell esds is used for estimating esds involving l.s. planes.
Refinement. Refinement of F^2^ against ALL reflections. The weighted R-factor wR and goodness of fit S are based on F^2^, conventional R-factors R are based on F, with F set to zero for negative F^2^. The threshold expression of F^2^ > 2sigma(F^2^) is used only for calculating R-factors(gt) etc. and is not relevant to the choice of reflections for refinement. R-factors based on F^2^ are statistically about twice as large as those based on F, and R- factors based on ALL data will be even larger.

### Fractional atomic coordinates and isotropic or equivalent isotropic displacement parameters (Å^2^)

**Table:**

	*x*	*y*	*z*	*U*_iso_*/*U*_eq_	
Br1	1.03493 (2)	0.63367 (3)	0.15480 (4)	0.06855 (15)	
Cl1	0.71461 (6)	1.15851 (6)	0.46918 (11)	0.0757 (3)	
N1	0.69113 (14)	0.83453 (15)	0.6106 (2)	0.0358 (4)	
N2	0.74414 (15)	0.76627 (15)	0.5225 (2)	0.0378 (4)	
O1	0.75662 (14)	0.64019 (12)	0.71573 (18)	0.0477 (4)	
C1	0.62315 (16)	1.00731 (17)	0.6291 (3)	0.0342 (5)	
C2	0.62851 (18)	1.11440 (19)	0.5940 (3)	0.0433 (6)	
C3	0.5678 (2)	1.1884 (2)	0.6563 (3)	0.0542 (7)	
H3	0.5732	1.2596	0.6310	0.065*	
C4	0.4996 (2)	1.1563 (2)	0.7553 (3)	0.0572 (7)	
H4	0.4581	1.2057	0.7970	0.069*	
C5	0.4924 (2)	1.0510 (2)	0.7933 (3)	0.0523 (7)	
H5	0.4459	1.0293	0.8607	0.063*	
C6	0.55369 (18)	0.9775 (2)	0.7319 (3)	0.0429 (6)	
H6	0.5487	0.9066	0.7596	0.052*	
C7	0.68317 (17)	0.92838 (18)	0.5560 (3)	0.0367 (5)	
H7	0.7159	0.9469	0.4686	0.044*	
C8	0.77228 (16)	0.67075 (17)	0.5825 (2)	0.0336 (5)	
C9	0.82605 (15)	0.60205 (17)	0.4763 (2)	0.0319 (5)	
C10	0.89265 (16)	0.64395 (18)	0.3775 (3)	0.0359 (5)	
H10	0.9036	0.7166	0.3742	0.043*	
C11	0.94190 (17)	0.5767 (2)	0.2851 (3)	0.0395 (5)	
C12	0.9267 (2)	0.4693 (2)	0.2864 (3)	0.0487 (6)	
H12	0.9603	0.4249	0.2225	0.058*	
C13	0.8606 (2)	0.4287 (2)	0.3844 (3)	0.0498 (6)	
H13	0.8490	0.3561	0.3859	0.060*	
C14	0.81123 (18)	0.49435 (18)	0.4804 (3)	0.0411 (5)	
H14	0.7679	0.4658	0.5479	0.049*	
H2	0.749 (2)	0.784 (2)	0.4206 (17)	0.080*	

### Atomic displacement parameters (Å^2^)

**Table:**

	*U*^11^	*U*^22^	*U*^33^	*U*^12^	*U*^13^	*U*^23^
Br1	0.0647 (2)	0.0867 (3)	0.0630 (2)	0.00759 (16)	0.03940 (15)	0.00891 (15)
Cl1	0.0682 (5)	0.0610 (5)	0.1056 (6)	0.0046 (4)	0.0393 (4)	0.0307 (4)
N1	0.0373 (10)	0.0395 (11)	0.0326 (9)	0.0065 (8)	0.0121 (8)	−0.0033 (8)
N2	0.0477 (11)	0.0400 (11)	0.0292 (9)	0.0105 (9)	0.0168 (8)	0.0004 (8)
O1	0.0696 (12)	0.0445 (10)	0.0339 (8)	0.0059 (8)	0.0236 (8)	0.0038 (7)
C1	0.0307 (11)	0.0393 (12)	0.0322 (11)	0.0033 (9)	0.0031 (9)	−0.0017 (9)
C2	0.0375 (12)	0.0439 (14)	0.0487 (14)	0.0022 (10)	0.0063 (10)	0.0019 (10)
C3	0.0551 (15)	0.0383 (14)	0.0686 (18)	0.0081 (12)	0.0063 (14)	−0.0028 (12)
C4	0.0551 (16)	0.0562 (17)	0.0609 (17)	0.0170 (13)	0.0102 (14)	−0.0140 (13)
C5	0.0474 (14)	0.0642 (18)	0.0484 (15)	0.0073 (13)	0.0175 (12)	−0.0042 (13)
C6	0.0429 (13)	0.0435 (14)	0.0443 (13)	0.0035 (11)	0.0127 (11)	−0.0016 (11)
C7	0.0383 (12)	0.0423 (14)	0.0314 (11)	0.0033 (10)	0.0114 (9)	0.0015 (10)
C8	0.0352 (11)	0.0369 (12)	0.0300 (11)	0.0002 (9)	0.0096 (9)	−0.0022 (9)
C9	0.0297 (11)	0.0382 (12)	0.0280 (10)	0.0051 (9)	0.0049 (8)	0.0002 (9)
C10	0.0369 (12)	0.0392 (12)	0.0329 (11)	0.0027 (10)	0.0088 (9)	−0.0006 (9)
C11	0.0341 (11)	0.0526 (15)	0.0336 (11)	0.0064 (10)	0.0112 (9)	0.0018 (10)
C12	0.0508 (14)	0.0518 (16)	0.0450 (14)	0.0149 (12)	0.0119 (11)	−0.0089 (12)
C13	0.0573 (15)	0.0353 (13)	0.0576 (15)	0.0054 (12)	0.0114 (12)	−0.0040 (11)
C14	0.0410 (13)	0.0413 (14)	0.0427 (13)	0.0021 (10)	0.0120 (10)	0.0025 (10)

### Geometric parameters (Å, °)

**Table:**

Br1—C11	1.892 (2)	C5—C6	1.376 (3)
Cl1—C2	1.738 (2)	C5—H5	0.93
N1—C7	1.270 (3)	C6—H6	0.93
N1—N2	1.386 (2)	C7—H7	0.93
N2—C8	1.339 (3)	C8—C9	1.491 (3)
N2—H2	0.893 (10)	C9—C14	1.376 (3)
O1—C8	1.226 (2)	C9—C10	1.392 (3)
C1—C2	1.388 (3)	C10—C11	1.372 (3)
C1—C6	1.393 (3)	C10—H10	0.93
C1—C7	1.459 (3)	C11—C12	1.372 (4)
C2—C3	1.378 (3)	C12—C13	1.376 (4)
C3—C4	1.366 (4)	C12—H12	0.93
C3—H3	0.93	C13—C14	1.379 (3)
C4—C5	1.374 (4)	C13—H13	0.93
C4—H4	0.93	C14—H14	0.93
			
C7—N1—N2	114.24 (17)	N1—C7—H7	119.7
C8—N2—N1	119.58 (17)	C1—C7—H7	119.7
C8—N2—H2	122 (2)	O1—C8—N2	123.53 (19)
N1—N2—H2	118 (2)	O1—C8—C9	121.02 (19)
C2—C1—C6	116.9 (2)	N2—C8—C9	115.44 (18)
C2—C1—C7	122.0 (2)	C14—C9—C10	119.6 (2)
C6—C1—C7	121.1 (2)	C14—C9—C8	118.69 (19)
C3—C2—C1	122.1 (2)	C10—C9—C8	121.7 (2)
C3—C2—Cl1	118.1 (2)	C11—C10—C9	119.2 (2)
C1—C2—Cl1	119.79 (18)	C11—C10—H10	120.4
C4—C3—C2	119.6 (2)	C9—C10—H10	120.4
C4—C3—H3	120.2	C10—C11—C12	121.8 (2)
C2—C3—H3	120.2	C10—C11—Br1	119.05 (18)
C3—C4—C5	120.1 (2)	C12—C11—Br1	119.17 (17)
C3—C4—H4	120.0	C11—C12—C13	118.6 (2)
C5—C4—H4	120.0	C11—C12—H12	120.7
C4—C5—C6	120.2 (2)	C13—C12—H12	120.7
C4—C5—H5	119.9	C12—C13—C14	120.8 (2)
C6—C5—H5	119.9	C12—C13—H13	119.6
C5—C6—C1	121.3 (2)	C14—C13—H13	119.6
C5—C6—H6	119.4	C9—C14—C13	120.0 (2)
C1—C6—H6	119.4	C9—C14—H14	120.0
N1—C7—C1	120.54 (19)	C13—C14—H14	120.0

### Hydrogen-bond geometry (Å, °)

**Table:**

*D*—H···*A*	*D*—H	H···*A*	*D*···*A*	*D*—H···*A*
N2—H2···O1^i^	0.89 (1)	1.98 (1)	2.854 (2)	165 (3)

Symmetry codes: (i) *x*, −*y*+3/2, *z*−1/2.
